# The diagnosis is obvious… or is it? A case of idiopathic segmental anhidrosis

**DOI:** 10.1016/j.clinme.2023.100009

**Published:** 2024-01-18

**Authors:** Shahzaib Rehan, Mohamed Ahamed

**Affiliations:** Royal Glamorgan Hospital, Ynysmaerdy, Pontyclun, Wales CF72 8XR, UK

**Keywords:** Medical education, Ophthalmology, Medicine, Acute eyecare, General medicine

## Case presentation

A 53-year-old man presented to his optometrist concerned about a 1-week history of asymmetry in his pupil sizes. His optometrist confirmed the presence of anisocoria (right pupil 2 mm and left eye 3 mm) and with an otherwise normal examination referred him into the hospital eye care setting urgently for a second opinion.

8 days later, he was reviewed by an ophthalmologist. The patient denied any symptoms, including headaches, diplopia and trauma. His past medical and ocular histories were unremarkable and he did not take any regular medication. He denied allergies and alcohol consumption. The patient commented that he had been aware of reduced/absent sweating on the right side of his face.

On examination his vision was noted to be right eye 6/5 and left eye 6/6. No anisocoria was noted, there was no ptosis, eye movements were full and ocular health assessment was unremarkable.

**Fig. 1:**
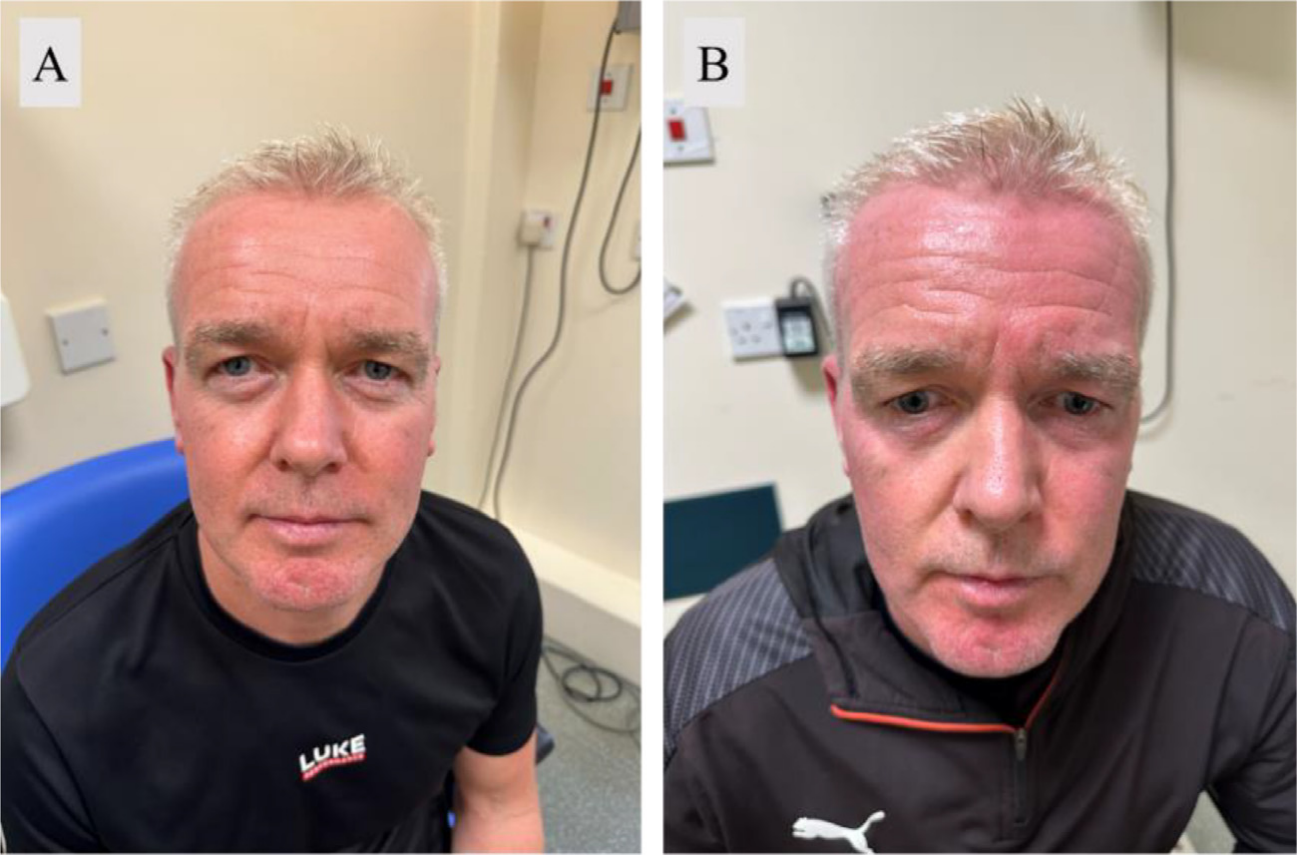
Photographs of the patient taken (a) pre-exercise and (b) post-exercise.

Pre- and post-exercise facial photographs were taken (Fig. 1). The anhidrosis and reduced erythema on the right side of his face was visible.

Interestingly the patient was worked up as a Horner's case but all of the investigations (including a MRI of his head and neck and CT of his head/neck/thorax) were unremarkable and so he was diagnosed with idiopathic segmental anhidrosis with physiological anisocoria.

## Discussion

Anhidrosis is the inability to sweat. In addition to Horner's syndrome, causes include peripheral alterations in the eccrine glands, medication and central or neuropathic disease, but cases can also be idiopathic.

To treat the patient, the underlying cause of the anhidrosis needs to be identified and addressed. Conservative lifestyle interventions such as water spray bottles to provide a cooling effect can be utilised.

Most doctors are aware of the link between anhidrosis and Horner's syndrome but the other differentials are less well known.

